# Recent advancements in the management of retinoblastoma and uveal melanoma

**DOI:** 10.12688/f1000research.11941.1

**Published:** 2018-04-18

**Authors:** Amy C Schefler, Ryan S Kim

**Affiliations:** 1 Retina Consultants of Houston, Houston, TX, 77030, USA; 2 Blanton Eye Institute, Houston Methodist Hospital, Houston, TX, 77030, USA; 3 McGovern Medical School, University of Texas Health Science Center at Houston, Houston, TX, 77030, USA

**Keywords:** retinoblastoma, uveal melanoma, ocular tumors

## Abstract

Retinoblastoma and uveal melanoma are the most common intraocular malignancies observed in pediatric and adult populations, respectively. For retinoblastoma, intra-arterial chemotherapy has dramatically improved treatment outcomes and eye salvage rates compared with traditional salvage rates of systemic chemotherapy and external beam radiation therapy. Intravitreal injections of chemotherapy have also demonstrated excellent efficacy for vitreous seeds. Uveal melanoma, on the other hand, is treated predominantly with iodine-125 plaque brachytherapy or with proton beam therapy. Major strides in uveal melanoma genomics have been made since the early 2000s, allowing ocular oncologists to better understand the metastatic risks of the tumor on the basis of specific genetic signatures. Loss-of-function mutations of the *BAP1* gene are associated with the highest metastatic risk, whereas gain-of-function mutations of *SF3B1 *and *EIF1AX* often confer a better prognosis. Expression of a cancer-testis antigen called PRAME (preferentially expressed antigen in melanoma) has been shown to increase metastatic risks in both low-risk and high-risk melanomas. New therapeutic approaches, including molecular therapies and nanoparticle phototherapy, are currently being investigated as alternative treatment modalities for uveal melanoma.

## Introduction

Retinoblastoma and uveal melanoma, albeit rare, are the most commonly observed intraocular malignancies in pediatric and adult populations, respectively. Retinoblastoma occurs during early childhood in 1 per 16,000 people worldwide ^1^, whereas uveal melanoma occurs on average in Caucasians in their fifties and sixties ^2^. Recent advancements in the techniques used to treat these two types of cancer have drastically enhanced patient outcomes and eye salvage rates. In this review, we briefly discuss current treatment guidelines and some emerging topics in the management of primary retinoblastoma and primary uveal melanoma.

## Retinoblastoma

Retinoblastoma presents unilaterally in approximately 60–70% of cases and bilaterally in the remaining 30–40%. In unilateral retinoblastoma, close to 90% of patients present with a sporadic mutation, whereas heritable mutations of the *RB1* gene (located at chromosome 13q14) with a known affected family member occur in approximately 10% ( Table 1) ^3, 4^. A small fraction of non-heritable retinoblastoma presents with a *MYCN* oncogene mutation that results in a unilateral, sporadic tumor ^5^. Unlike unilateral disease, bilateral retinoblastoma is always due to a germline mutation and commonly presents earlier in life than unilateral cases. With the advent of improved sequencing techniques, mosaicism is increasingly being recognized in both unilateral and bilateral patients. The presence of a germline *RB1* mutation increases the risk for secondary cancers, especially when retinoblastoma is treated with external beam radiation (EBR) ^6^.

**Table 1.** Comparison of characteristics between sporadic and hereditary retinoblastoma.

	Sporadic	Germline	Germline- mosaic
Number of mutated cells	One	All	Variable
Laterality	Always unilateral	85% bilateral, 15% unilateral	Either unilateral or bilateral
Age of onset	18–24 months	12–18 months	Variable
Chance of inheritance to offspring	0%	45%	Variable

Both the Reese–Ellsworth and the International Classification of Retinoblastoma (ICRB) systems can be used to classify retinoblastoma, although the latter, newer system has been widely adopted in the last decade ( Table 2). ICRB divides retinoblastoma into five categories; class A is the least advanced and E is the most advanced type ^7^. Focal therapies such as laser ablation and cryotherapy can be used for retinoblastoma with ICRB classes A and B, whereas more advanced cases (ICRB class C, D, or E) are preferentially treated with systemic chemotherapy or intra-arterial chemotherapy (IAC) over EBR or plaque brachytherapy because of their adverse effects. Enucleation of the eye is performed when there is a potential risk of extraocular extension, especially in class E eyes, or when all prior treatments have failed.

**Table 2.** Summary of Reese–Ellsworth and International Classification of Retinoblastoma (ICRB) classification systems.

Reese–Ellsworth classification	International Classification of Retinoblastoma
Group 1 - 1a: solitary tumor less than 4 disc diameter (DD) at or behind equator - 1b: multiple tumors all under 4 DD at or behind equator	Group A: tumors <3 mm and away from fovea and optic disc
Group 2 - 2a: solitary tumor 4–10 DD at or behind equator - 2b: multiple tumors 4–10 DD at or behind equator	Group B: tumors >3 mm, located at macula/peripapillary region, or with subretinal fluid
Group 3 - 3a: tumors anterior to equator - 3b: solitary tumor >10 DD behind the equator	Group C: tumors with focal vitreous or subretinal seeds within 3 mm of tumor
Group 4 - 4a: multiple tumors with some >10 DD - 4b: any tumor extending to ora serrata	Group D: tumors with diffuse vitreous or subretinal seeds >3 mm away from tumor
Group 5 - 5a: tumors involving >50% of retina - 5b: tumors with vitreous seeding	Group E: tumors covering >50% of globe with or without neovascular glaucoma, hemorrhage, extension of tumor to optic nerve/anterior chamber

### Intra-arterial chemotherapy as primary treatment

**Figure 1.**
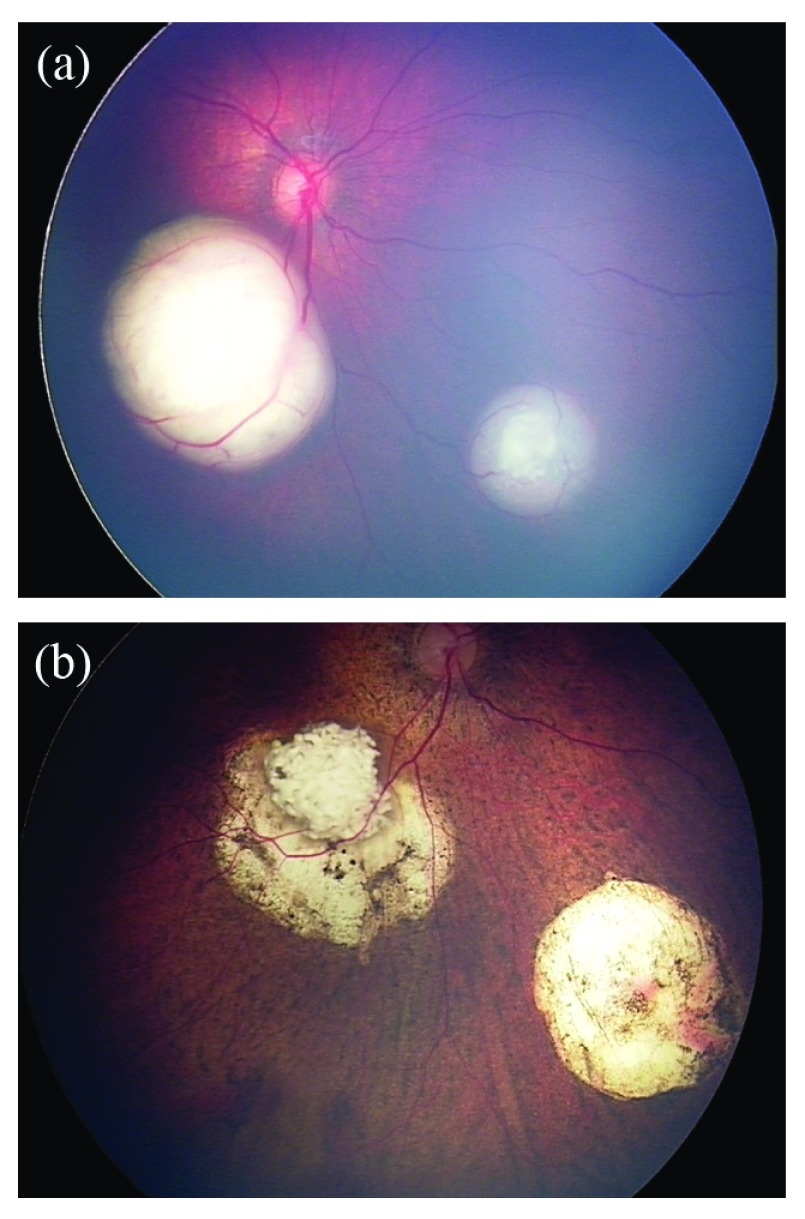
Retinoblastoma of a 5-month-old patient before and after intra-arterial chemotherapy (IAC). ( **a**) Fundus photograph of the right eye before IAC, demonstrating macular and inferonasal lesions. ( **b**) Fundus photograph of the same eye 13 months after the initial IAC treatment. The patient underwent three IAC cycles and adjuvant therapy, including five sessions of laser ablation and two sessions of cryotherapy.

EBR was used as primary therapy for retinoblastoma in most cases until the early 1990s and then intravenous chemotherapy (IVC) until the early 2000s. In 2004, a group of Japanese investigators reported a new technique of balloon-occluding the internal carotid artery distal to the ostium of the ophthalmic artery and then locally injecting melphalan to treat retinoblastoma ^8^. In 2008, Abramson *et al*. reported a more sophisticated technique of directly infusing melphalan into the ophthalmic artery with a microcatheter that many centers have now adopted with variations ^9^. In this report, seven out of 10 eyes classified as Reese–Ellsworth V and originally scheduled for enucleation were salvaged by IAC. Numerous studies have since reported on the efficacy of IAC compared with that of IVC. In one study by Shields *et al*. ^10^, global salvage rates for IAC and IVC for class D tumors were 91% and 48%, respectively, demonstrating that IAC can be particularly successful at treating more advanced tumors. Therefore, at many large centers of excellence, IAC is the preferred treatment modality for unilateral and non-hereditary retinoblastoma. Bilateral retinoblastoma with a germline *RB1* mutation can be treated with either systemic chemotherapy or tandem IAC, in which IAC is performed in both eyes in a single IAC session ^11^. In case the ophthalmic artery anatomy is not amenable to IAC, the external carotid artery can be alternately used to gain access to the ocular vasculature ^12^. Most large centers have reported superior ocular salvage rates with IAC compared with systemic chemotherapy ( Figure 1). Systemic treatment-related immediate effects such as immunosuppression are also rarer with IAC ^13, 14^. Clinicians at centers that continue to use systemic chemotherapy have reported concerns about increased risks of metastatic retinoblastoma and risks of secondary cancers ^15, 16^. However, these controversies are unresolved with both fierce advocates and staunch opponents of IAC in existence with no clear sign of a definitive multi-center collaborative trial in the works that might settle the debate. As such, there continues to be a heterogeneity of treatment approaches in the US and abroad.

In many centers, IAC has been widely adopted as the primary therapy for retinoblastoma, and numerous publications have reported successful treatment outcomes. One of the recent reports, by Abramson *et al*., demonstrated that over 90% of patients now undergo IAC for primary therapy, and the global salvage rate at 48 months post-IAC significantly increased, from 68% in the late 2000s to 92.7% between 2010 and 2014 ^17^. Shields *et al*. reported 100% ocular salvage for ICRB class B and C eyes, for which IAC was used as primary treatment ^18^. IAC has also been demonstrated to be highly efficacious for advanced tumors, and 5-year ocular survival exceeded 70% for class D tumors ^18– 20^. Class E tumors show mixed results: ocular salvage ranged from 30% to 70% in the literature ^18– 20^.

### Age and weight threshold for intra-arterial chemotherapy

Whereas the safety and efficacy of IAC have been well demonstrated in multiple studies over the past decade, the guideline for age and weight threshold for IAC has not yet been strictly defined. It has generally been assumed that IAC should be reserved until patients with retinoblastoma reach a weight of 10 kg or the equivalent age because of potential procedural complications, such as groin hematomas or femoral artery dissection ^21^. However, several studies have recently demonstrated that patients with retinoblastoma who are younger than 3 months of age can be successfully treated with IAC ^22, 23^. The safety of IAC can be further enhanced with ultrasound guidance for femoral artery catheterization, which has long been used in various kinds of pediatric procedures. A pilot study of six patients with retinoblastoma ^24^ demonstrated no procedural complications when IAC was administered with ultrasound guidance to patients with a median weight of 9.2 kg at the first IAC cycle. Recent literature suggests that younger and treatment-naïve patients may achieve better oncologic efficacy when they receive a minimal number of IAC cycles ^25, 26^. Also, Gobin *et al*. reported that eyes that received IAC as primary treatment had an ocular event-free survival rate of 81.7% after 2 years, which was significantly higher than the rate of 58.4% for the eyes that had undergone IVC or EBR prior to IAC ^27^. Therefore, unless there are known contraindications for IAC, such as metastatic retinoblastoma, optimal treatment outcomes may be achieved when the patients undergo IAC at the earliest age possible.

### Intravitreal melphalan for treating vitreous seeds

Vitreous seeds are groups of tumor cells that break off from the primary lesion and are commonly seen in advanced retinoblastoma. Vitreous seeds are seen in ICRB class C, D, and E. Dust, spheres, and clouds are the three forms of vitreous seeds with different patterns of response to chemotherapy which have been previously characterized ^28^. IAC has limited efficacy for vitreous seeds because of the avascular nature of the vitreous. Intravitreal injections of chemotherapy via the pars plana have now been widely adopted for persistent or recurrent vitreous seeds after primary treatment with IAC or systemic chemotherapy ^28– 30^. In general, intravitreal melphalan or topotecan is combined with a simultaneous treatment of the retinal tumors from which the vitreous seeds originate. To prevent any potential extension of tumor seeds via the needle tract, clinicians typically take various safety measures, including cryotherapy applied to the needle site, visualization of the pars plana with ultrasound biomicroscopy, washing of the ocular surface, and subconjunctival chemotherapy ^30, 31^. The safety and efficacy of intravitreal injection of melphalan have been demonstrated in multiple studies ^28, 32^.

### Intravenous chemotherapy

IVC has been used as primary and secondary therapy for retinoblastoma for over two decades. A three-agent combination (carboplatin, vincristine, and etoposide) is commonly used ^33^, and other agents, including topotecan or cisplatin, can be additionally administered depending on the patient’s response to the agents ^34^. Multiple studies report that over 90% of tumor control was achieved by using IVC, especially for ICRB classes A, B, and C ^10, 35^. IVC can be effective at managing both bilateral and germline retinoblastoma. Some investigators have reported that IVC helps in preventing extraocular secondary cancers, including trilateral retinoblastoma in which extraocular cancer occurs in the pineal or suprasellar region ^36– 38^; however, this is controversial. There are some rare but recognized side effects of IVC, including neurotoxicity, immunosuppression, secondary leukemia, nephrotoxicity, and ototoxicity ^34^.

### Radioactive plaque for persistent, recurrent retinoblastoma

Owing to concerns for radiation retinopathy, radioactive plaque brachytherapy is most commonly used as rescue therapy for relatively small solitary tumors in ICRB class A or B ^39, 40^. However, plaque brachytherapy is one of the preferred secondary treatment modalities before enucleation. There have been published studies that demonstrated the efficacy of iodine-125 plaque brachytherapy as salvage treatment for retinoblastoma after both IAC and IVC ^39, 40^.

## Uveal melanoma

Uveal melanoma is a malignant cancer that occurs in 4.9 people per million in the US alone ^41^. As the name suggests, uveal melanoma can occur in any part of the uveal tract, including the iris, ciliary body, or choroid, and the involvement of the choroid is the most common. Uveal melanoma is known to spread hematogenously, and the most common sites of metastasis in descending order are liver, lung, and bone ^42^. Mean overall 5-year survival rate has remained stable at approximately 80% over the past several decades ^2, 43^, while the 5-year survival rate drastically decreases once the tumor metastasizes. Lifetime rates of metastases in patients with uveal melanoma are controversial, but rates reported in the scientific literature range from 25% to 50% with a median survival of 6 months to a year after the development of metastatic disease ^44– 46^. Some studies have reported a longer median survival once metastatic disease is diagnosed, but other authors claim that lead-time bias explains these results ^47^. Upon initial diagnosis, most patients currently receive plaque brachytherapy, proton beam therapy, or enucleation, except for some iris tumors that can be surgically resected.

### Genetic and histopathologic analyses of uveal melanoma

Uveal melanoma is largely due to sporadic mutations in uveal melanocytes, and inherited germline mutations that contribute to the development of this tumor are extremely rare, occurring in 3% to 4% of patients ^48^. However, a number of publications since the early 1990s have discussed the importance of cytogenetic changes of the cancer cells which significantly affect the prognosis. Uveal melanoma is histopathologically characterized by spindle and epithelioid cells ^49^. Standard cytology procedures, including cell block analysis with hematoxylin–eosin stain and HMB45/Ki67 immunohistochemical stain, can identify cells acquired from biopsies. Although epithelioid cells are strongly associated with more aggressive behavior, most uveal melanomas contain mixed spindle and epithelioid cells regardless of the predisposed metastatic risk ^49^. One study published that epithelioid and necrotic cell types have a statistically significantly higher rate of 5-year metastatic mortality rate than other cell-type findings ^50^. In the same study, cytopathologic classification was found to be an independent prognostic factor for metastatic death ^50^.

In the early 1990s, Prescher *et al*. first reported that monosomy 3, the abnormal presence of only one copy of chromosome 3, was a commonly observed cytogenetic abnormality in uveal melanoma ^51^. Since then, a number of studies focusing on the genetics of uveal melanoma have been published. Several key driver genes, including *GNA11*, *GNAQ*, *BAP1*, *SF3B1*, and *EIF1AX*, have been identified to be involved in the development and metastasis of the cancer ** ^52^. Combinations of mutations of these genes lead to variations in the development and metastasis of uveal melanoma. Of these, *GNAQ* and *GNA11* mutations are involved in the early stage of oncogenesis and occur in a mutually exclusive manner in approximately 91% of the patients ^53^. Because these mutations occur early in oncogenesis, neither one confers valuable prognostic information. Recently, a loss-of-function mutation of *BAP1*, a tumor suppressor gene, was discovered to be heavily associated with more malignant types of uveal melanoma. Loss of *BAP1* induces dedifferentiation of melanoma cells and the development of stem cell-like characteristics ^54, 55^. On the other hand, hemizygous, gain-of-function mutations of *SF3B1* and *EIF1AX* generally indicate a better prognosis and occur in lower-risk melanomas ^56^. Of note, melanomas with *SF3B1* mutations are associated with late-onset metastases ^57^. *BAP1*, *SF3B1*, and *EIF1AX* mutations mostly occur late in tumor development and also occur in a mutually exclusive fashion ^58^.

Gene expression profile (GEP) analysis and multiplex ligand-dependent probe amplification (MLPA) have been adopted by ocular oncologists to elucidate each tumor’s genetic characteristics ^59, 60^. GEP testing uses a polymerase chain reaction (PCR)-based 15-gene panel and classifies uveal melanoma as either class 1 (low risk for metastasis) or class 2 (high risk for metastasis) ^59, 61^. Class 1 is further divided into 1A and 1B; 1A tumors remain relatively low-risk for metastasis, whereas the risk of metastasizing in 1B appears to be higher than the 1A group over time. The 5-year published metastatic rates for class 1A, 1B, and 2 tumors are 2%, 21%, and 72%, respectively ^58^. It has been observed that class 1B uveal melanoma, though categorized under class 1, behaves more similarly to class 2 tumors and therefore requires close monitoring for progression to metastasis. *BAP1* somatic mutations are observed predominantly in class 2 tumors, whereas *SF3B1* or *EIF1AX* mutations are seen more frequently in class 1 tumors ^54^. It is reported that *BAP1* mutations can be observed in approximately 80% of metastatic uveal melanoma cells ^54^. In another study, 71%, 11%, and 0% of patients with primary uveal melanoma who developed metastases carried *BAP1*, *SF3B1*, and *EIF1AX* mutations, respectively, signifying that *EIF1AX* and *SF3B1* mutations generally confer a good prognosis ^62^. In the largest single-institution case series of over 1,000 patients, 3-year Kaplan–Meier estimates for metastatic uveal melanoma were provided for the following cytogenetic abnormalities: 5% for partial monosomy of chromosome 3; 19% for complete monosomy 3; 23% for loss of 6q; 29% for loss of 8p; 21% for gain of 8q; 1% for disomy of 3, 6, and 8; 29% for complete monosomy 3, 6p gain, and 8q gain; 14% for complete monosomy of 3, disomy of 6, and gain of 8q and 8p; 27% for complete monosomy of 3, disomy of 6, and gain of 8q; and 28% for complete monosomy of 3, disomy of 6, gain of 8q, and loss of 8p ^63^.

Recently, scientists have discovered that certain uveal melanomas that express a cancer-testis antigen called preferentially expressed antigen in melanoma (PRAME) are closely associated with an increased risk of metastasis in both class 1 and 2 uveal melanomas ^64, 65^. Also, class 1 tumors that are PRAME ^+^ were found to be associated with *SF3B1* mutations and inversely to *EIF1AX* mutations ^65^. A combination of *SF3B1* mutations and PRAME expression appears to contribute to late metastases in class 1 tumors ^66^, while PRAME ^+^ class 2 tumors exhibited accelerated progression to metastases ^65^. PRAME is currently being investigated as a potential target for immunotherapy in primary and metastatic uveal melanoma ^67^. The Collaborative Ocular Oncology Group 2 (COOG2) is a currently enrolling multi-center prospective clinical trial in which PRAME genomics will be examined along with long-term clinical outcomes.

### Fine needle aspiration biopsy

As research in melanoma genomics has grown explosively, safe and adequate acquisition of tumor cells has become increasingly important for both clinical and research purposes. Fine needle aspiration biopsy (FNAB) is performed by using small-sized needles (23-, 25-, or 27-gauge) or vitrectomy probes in either a transvitreal or a trans-scleral manner, depending on the tumor location. For tumors that are anterior to the equator with direct access to the needle, the trans-scleral method is typically chosen. For posterior tumors that are more difficult to access via a trans-scleral biopsy, transvitreal biopsy can be performed by using indirect ophthalmoscopy or standard retinal instrumentation, including chandelier lighting that gives direct visualization, and valved trocars, which serve to maintain the intraocular pressure during biopsy and prevent the tracking of tumor cells along the needle tract. After the aspiration of tumor cells, cryotherapy is applied to the needle insertion site in order to prevent any iatrogenic extraocular extension of tumor cells via the needle tract. Safety of FNAB for uveal melanoma was recently reaffirmed in a prospective, *in vivo* study ^68^. Many studies have demonstrated high cellular yield rates, ranging from 68% to over 90%, for cytopathologic and genomic analyses ^69– 73^.

### Plaque brachytherapy

The Collaborative Ocular Melanoma Study (COMS) found no statistically significant difference in survival between patients who underwent plaque brachytherapy and patients who underwent enucleation ^74^. Since then, most centers have adopted plaque brachytherapy as the standard treatment for uveal melanoma. Multiple types of isotopes are used for ophthalmic brachytherapy. In the US, ^125^I is the most frequently used radioisotope after the COMS study, whereas ^106^Ru and ^103^Pd are more commonly used in Europe and other countries ^75^. ^125^I and ^103^Pd both emit low-energy gamma rays and thus cause less damage to surrounding healthy tissues compared with isotopes that were used in the first half of the 20th century, such as ^60^Co. ^106^Ru, on the other hand, emits beta rays and has a quicker dose fall-off ^75^. The steeper the dose gradient, the more concentrated the radiation effect on the basal side of the tumor and conversely less radiation toward the apex. ^106^Ru has an advantage of less radiation effect to other ocular structures compared with that of ^125^I or ^103^Pd.

**Figure 2.**
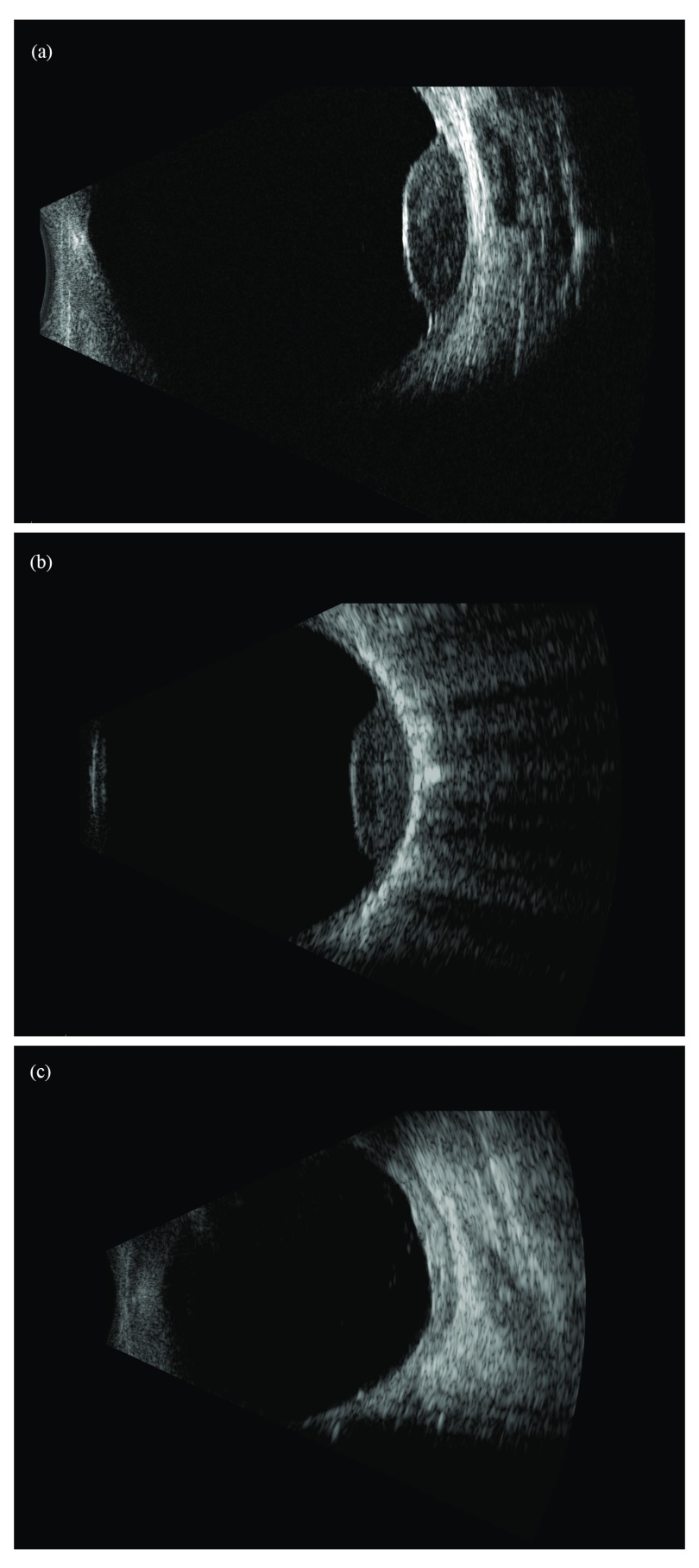
Uveal melanoma of a 66-year-old patient before and after plaque brachytherapy. ( **a**) B-scan ultrasound image of the right eye before the plaque implantation. ( **b**) B-scan ultrasound image of the same eye intraoperatively, demonstrating full coverage of the tumor with the plaque. ( **c**) B-scan ultrasound image of the same eye 3 years after the plaque therapy, demonstrating regression of the tumor.

Multiple studies have demonstrated that plaque therapy and enucleation result in comparable mortality rates over 20 years of follow-up ^76^. Iodine-125 brachytherapy has become the most commonly used treatment modality for uveal melanoma in the US with excellent clinical outcomes ( Figure 2).

Local recurrence of tumor cells at the ocular site is a critical complication to be avoided after plaque therapy. Multiple studies have demonstrated that the likelihood of metastasis increases dramatically after local recurrence occurs ^77, 78^. However, the 5-year local recurrence rate has steadily decreased from 10.3% at the time of the COMS ^76^ to 2.4% to approximately 4.7% ^77, 79^ over the past two decades. Indeed, a recent publication that reported preliminary clinical outcomes with a median follow-up of 21.6 months ^80^ demonstrated zero local recurrence, which may be attributed to several factors. First, newer plaque designs ^81, 82^ are thinner than the traditional COMS plaques and are customized to conform better to each patient’s eye, leading to better coverage of the tumor and less radiation scatter outside the targeted area. Second, intraoperative ultrasonographic confirmation of plaque positioning, which has been used more over the past decade, ensures precise placement of the plaque ^83– 85^. A recent study at the Cleveland Clinic reported that plaque treatment failure decreased from 9.3% to 1.5% since intraoperative ultrasound was adopted ^86^. Intraoperative transillumination of the tumor and preoperative 3D planning with Plaque Simulation software can further enhance the accuracy of plaque placement ^80, 87^. Treatment outcomes of plaque brachytherapy, including 5-year mortality and local recurrence rates (4%), are comparable to those of proton beam radiotherapy in recent publications ^88, 89^. Both plaque and proton beam therapy are known to cause ocular complications, including cataracts, radiation retinopathy, and radiation optic neuropathy. The COMS demonstrated that nearly 50% of patients who receive plaque brachytherapy had significant vision loss by 3 years post-treatment because of these complications ^90^.

### Clinical features with prognostic significance

In addition to the fact that GEP class 2 melanomas have higher mortality rates than GEP class 1 tumors, several additional factors that contribute valuable prognostic information have recently been identified. Correa and Augsburger recently reported that the largest basal diameter (LBD) of the tumor can serve as an independent prognostic factor for metastasis and metastatic death ^91^. Harbour *et al*. reported that class 2 tumors with an LBD over 12 mm had a significantly lower 5-year metastasis-free survival ^92^. Also, increased patient age, larger tumor apical height, and ciliary body involvement of the tumor are associated with metastatic risk ^93, 94^. Traditionally, tumors with more malignant characteristics, such as tumors with monosomy 3 or those that metastasized, were reported to regress faster after plaque therapy ^95, 96^. In recent studies, the relationship between GEP class and the tumor regression rate after brachytherapy has been controversial. Whereas several studies ^97, 98^ found neither GEP class to be significantly associated with regression rate, another study reported that class 1 tumors regress faster ^99^. In the largest multi-center, retrospective cohort study that was recently published, investigators also reported that class 1 tumors regress at a statistically significantly faster rate than class 2 tumors after plaque radiation ^100^. Future multi-center studies will help elucidate a clearer relationship between GEP class and therapeutic response to radiation.

### Molecular therapies

Some recent studies have identified several key molecular pathways associated with specific genetic mutations. For example, the *BAP1* gene is known to regulate histone H2A function by removing ubiquitin molecules. When the *BAP1* gene is mutated, proper removal of ubiquitin from H2A is inhibited, leading to a dedifferentiated state of melanoma cells ^101^. Also, *GNAQ* and *GNA11* genes are closely related to transmembrane cell signaling ^52^. Activation mutation of *GNAQ* or *GNA11* keeps guanine nucleotide-binding proteins in an active state, which subsequently upregulates protein kinase C and mitogen-activated protein kinase pathways that are involved in the proliferation and differentiation of cells at the early stages of uveal melanoma oncogenesis ^102^. Many ongoing clinical trials (some of which are still accruing patients and some of which are now closed to new patient enrollment) are examining immunotherapy agents that target these pathways as well as several others for both high-risk and metastatic uveal melanoma ( Table 3 and Table 4).

**Table 3.** List of ongoing clinical trials of adjuvant molecular therapy for high-risk uveal melanoma.

ClinicalTrials.gov identifier	Study locations	Study title
NCT02223819	Columbia University, New York, NY Mount Sinai Comprehensive Cancer Center, Miami Beach, FL Memorial Sloan Kettering Cancer Center, New York, NY The Ohio State University, Columbus, OH	Crizotinib in High-Risk Uveal Melanoma Following Definitive Therapy
NCT02068586	Thomas Jefferson University, Philadelphia, PA	Adjuvant Sunitinib or Valproic Acid in High-Risk Patients With Uveal Melanoma

**Table 4.** List of ongoing clinical trials of molecular therapy for metastatic uveal melanomas.

ClinicalTrials.gov identifier	Study locations	Study title
NCT01979523	Moffitt Cancer Center, Tampa, FL Emory University/Winship Cancer Institute, Atlanta, GA Columbia University/Herbert Irving Cancer Center, New York, NY Memorial Sloan Kettering Cancer Center, New York, NY Vanderbilt University/Ingram Cancer Center, Nashville, TN MD Anderson Cancer Center, Houston, TX Institut Curie Paris, Paris, France The University of Liverpool, Liverpool, UK	Trametinib With or Without GSK2141795 in Treating Patients With Metastatic Uveal Melanoma
NCT01585194	University of Texas MD Anderson Cancer Center, Houston, TX	Phase II Study of Nivolumab in Combination With Ipilimumab for Uveal Melanoma
NCT02570308	Washington University, School of Medicine, St. Louis, MO Columbia University Medical Center – The New York Presbyterian Hospital, New York, NY Thomas Jefferson University Medical Oncology Clinic, Philadelphia, PA The Clatterbridge Cancer Centre, Wirral, Merseyside, UK Mount Vernon Cancer Centre, Northwood, Middlesex, UK	A Study of the Intra-Patient Escalation Dosing Regimen With IMCgp100 in Patients With Advanced Uveal Melanoma
NCT02359851	University of Chicago, Chicago, IL Vanderbilt-Ingram Cancer Center, Nashville, TN	Pembrolizumab in Treating Patients With Advanced Uveal Melanoma
NCT02273219	Bascom Palmer Eye Institute of University Of Miami Medical Center, Miami, FL Columbia University Medical Center, New York, NY Memorial Sloan Kettering Cancer Center, New York, NY	Trial of AEB071 in Combination With BYL719 in Patients With Melanoma
NCT01473004	Thomas Jefferson University, Philadelphia, PA	SIR-Spheres 90Y Microspheres Treatment of Uveal Melanoma Metastasized to Liver
NCT02678572	32 locations in the US, Austria, Belgium, France, Germany, Italy, Spain, Switzerland, and the UK	Percutaneous Hepatic Perfusion vs Best Alternative Care in Patients With Hepatic- dominant Ocular Melanoma (FOCUS)
NCT01814046	National Institutes of Health Clinical Center, Bethesda, MD	Immunotherapy Using Tumor Infiltrating Lymphocytes for Patients With Metastatic Ocular Melanoma

### Nanoparticle therapy

Nanoparticle therapy is an emerging cancer therapy, in which photosensitive nanoparticles preferentially bind tumor cells, followed by light activation of the nanoparticles ^103^. This is a minimally invasive yet highly specific treatment modality that can kill tumor cells with minimal damage to the surrounding normal tissues. For uveal melanoma, a phase 1b clinical trial has begun to investigate the safety of a new nanoparticle phototherapy for small to medium-sized tumors in 12 patients ( http://www.aurabiosciences.com/news-archive/2017/3/30/aura-biosciences-announces-initiation-of-phase-1b-clinical-trial-and-receipt-of-fda-fast-track-designation-for-au-011-for-the-treatment-of-primary-ocular-melanoma). Viral nanoparticle conjugates attach to the uveal melanoma cell membrane. When activated by a 589 nm laser, the particles selectively break down the tumor cell membrane without affecting adjacent tissues. This treatment modality, if proven successful in clinical trials, has the potential to preserve much of the patient’s vision and could be particularly groundbreaking in patients with small tumors that are close to critical ocular structures such as the optic nerve and the macula. The effect on rates of metastatic disease are still unknown.

## Conclusions

Extensive advancements have been made in the understanding and treatment of retinoblastoma and uveal melanoma over the past decade. Further knowledge of intraocular cancer genetics will lead to new clinical breakthroughs that will allow us to save more eyes and lives.

## References

[1] Dimaras H Corson TW Cobrinik D: Retinoblastoma. *Nat Rev Dis Primers.* 2015;1:15021. 10.1038/nrdp.2015.21 27189421 PMC5744255

[2] Singh AD Turell ME Topham AK: Uveal melanoma: trends in incidence, treatment, and survival. *Ophthalmology.* 2011;118(9):1881–5. 10.1016/j.ophtha.2011.01.040 21704381

[3] Chantada G Schaiquevich P: Management of retinoblastoma in children: current status. *Paediatr Drugs.* 2015;17(3):185–98. 10.1007/s40272-015-0121-9 25742925

[4] Abramson DH Schefler AC: Update on retinoblastoma. *Retina.* 2004;24(6):828–48. 15579980 10.1097/00006982-200412000-00002

[5] Rushlow DE Mol BM Kennett JY: Characterisation of retinoblastomas without *RB1* mutations: genomic, gene expression, and clinical studies. *Lancet Oncol.* 2013;14(4):327–34. 10.1016/S1470-2045(13)70045-7 23498719

[6] Moll AC Imhof SM Schouten-Van Meeteren AY: Second primary tumors in hereditary retinoblastoma: a register-based study, 1945–1997: is there an age effect on radiation-related risk? *Ophthalmology.* 2001;108(6):1109–14. 10.1016/S0161-6420(01)00562-0 11382638

[7] Linn Murphree A: Intraocular retinoblastoma: the case for a new group classification. *Ophthalmol Clin North Am.* 2005;18(1):41–53, viii. 10.1016/j.ohc.2004.11.003 15763190

[8] Yamane T Kaneko A Mohri M: The technique of ophthalmic arterial infusion therapy for patients with intraocular retinoblastoma. *Int J Clin Oncol.* 2004;9(2):69–73. 10.1007/s10147-004-0392-6 15108036

[9] Abramson DH Dunkel IJ Brodie SE: A phase I/II study of direct intraarterial (ophthalmic artery) chemotherapy with melphalan for intraocular retinoblastoma initial results. *Ophthalmology.* 2008;115(8):1398–404, 1404.e1. 10.1016/j.ophtha.2007.12.014 18342944

[10] Shields CL Jorge R Say EA: Unilateral Retinoblastoma Managed With Intravenous Chemotherapy Versus Intra-Arterial Chemotherapy. Outcomes Based on the International Classification of Retinoblastoma. *Asia Pac J Ophthalmol (Phila).* 2016;5(2):97–103. 10.1097/APO.0000000000000172 26765038

[11] Abramson DH Marr BP Francis JH: Simultaneous Bilateral Ophthalmic Artery Chemosurgery for Bilateral Retinoblastoma (Tandem Therapy). *PLoS One.* 2016;11(6):e0156806. 10.1371/journal.pone.0156806 27258771 PMC4892546

[12] Abruzzo TA Geller JI Kimbrough DA: Adjunctive techniques for optimization of ocular hemodynamics in children undergoing ophthalmic artery infusion chemotherapy. *J Neurointerv Surg.* 2015;7(10):770–6. 10.1136/neurintsurg-2014-011295 25179634

[13] Schefler AC Kleinerman RA Abramson DH: Genes and environment: effects on the development of second malignancies in retinoblastoma survivors. *Expert Rev Ophthalmol.* 2008;3(1):51–61. 10.1586/17469899.3.1.51 24904684 PMC4043380

[14] Michaels ST Abruzzo TA Augsburger JJ: Selective Ophthalmic Artery Infusion Chemotherapy for Advanced Intraocular Retinoblastoma: CCHMC Early Experience. *J Pediatr Hematol Oncol.* 2016;38(1):65–9. 10.1097/MPH.0000000000000471 26583615

[15] Ramasubramanian A Kytasty C Meadows AT: Incidence of pineal gland cyst and pineoblastoma in children with retinoblastoma during the chemoreduction era. *Am J Ophthalmol.* 2013;156(4):825–9. 10.1016/j.ajo.2013.05.023 23876864

[16] Yousef YA Soliman SE Astudillo PP: Intra-arterial Chemotherapy for Retinoblastoma: A Systematic Review. *JAMA Ophthalmol.* 2016;134(5):584–591. 10.1001/jamaophthalmol.2016.0244 26986443

[17] Abramson DH Fabius AW Issa R: Advanced Unilateral Retinoblastoma: The Impact of Ophthalmic Artery Chemosurgery on Enucleation Rate and Patient Survival at MSKCC. *PLoS One.* 2015;10(12):e0145436. 10.1371/journal.pone.0145436 26709699 PMC4692433

[18] Shields CL Manjandavida FP Lally SE: Intra-arterial chemotherapy for retinoblastoma in 70 eyes: outcomes based on the international classification of retinoblastoma. *Ophthalmology.* 2014;121(7):1453–60. 10.1016/j.ophtha.2014.01.026 24656794

[19] Abramson DH Fabius AW Francis JH: Ophthalmic artery chemosurgery for eyes with advanced retinoblastoma. *Ophthalmic Genet.* 2017;38(1):16–21. 10.1080/13816810.2016.1244695 28095092 PMC5475401

[20] Chen M Jiang H Zhang J: Outcome of intra-arterial chemotherapy for retinoblastoma and its influencing factors: a retrospective study. *Acta Ophthalmol.* 2017;95(6):613–8. 10.1111/aos.13333 27874261

[21] Gobin YP Dunkel IJ Marr BP: Combined, sequential intravenous and intra-arterial chemotherapy (bridge chemotherapy) for young infants with retinoblastoma. *PLoS One.* 2012;7(9):e44322. 10.1371/journal.pone.0044322 23028521 PMC3445577

[22] Chen M Zhao J Xia J: Intra-Arterial Chemotherapy as Primary Therapy for Retinoblastoma in Infants Less than 3 Months of Age: A Series of 10 Case-Studies. *PLoS One.* 2016;11(8):e0160873. 10.1371/journal.pone.0160873 27504917 PMC4978489

[23] Magan T Khoo CT Jabbour PM: Intraarterial Chemotherapy for Retinoblastoma in A 2-Month-Old Infant. *Retin Cases Brief Rep.* 2017;11(1):24–26. 10.1097/ICB.0000000000000279 26756523

[24] Kim RS Dannenbaum MJ Lin MW: Use of Femoral Artery Ultrasound During Intraarterial Chemotherapy for Children Under 10 kg With Retinoblastoma. *Retina.* 2017. 10.1097/IAE.0000000000001713 28541962

[25] Shields CL Kaliki S Shah SU: Minimal exposure (one or two cycles) of intra-arterial chemotherapy in the management of retinoblastoma. *Ophthalmology.* 2012;119(1):188–92. 10.1016/j.ophtha.2011.06.036 21975042

[26] Dillon AB Douglass A Jabbour P: Minimal exposure intra-arterial chemotherapy for children with retinoblastoma and 13q syndrome. *Oman J Ophthalmol.* 2016;9(3):164–166. 10.4103/0974-620X.192278 27843232 PMC5084500

[27] Gobin YP Dunkel IJ Marr BP: Intra-arterial chemotherapy for the management of retinoblastoma: four-year experience. *Arch Ophthalmol.* 2011;129(6):732–7. 10.1001/archophthalmol.2011.5 21320950

[28] Munier FL: Classification and management of seeds in retinoblastoma. Ellsworth Lecture Ghent August 24th 2013. *Ophthalmic Genet.* 2014;35(4):193–207. 10.3109/13816810.2014.973045 25321846 PMC4245997

[29] Ghassemi F Shields CL: Intravitreal melphalan for refractory or recurrent vitreous seeding from retinoblastoma. *Arch Ophthalmol.* 2012;130(10):1268–71. 10.1001/archophthalmol.2012.1983 23044940

[30] Munier FL Gaillard MC Balmer A: Intravitreal chemotherapy for vitreous seeding in retinoblastoma: Recent advances and perspectives. *Saudi J Ophthalmol.* 2013;27(3):147–50. 10.1016/j.sjopt.2013.06.003 24227979 PMC3770220

[31] Francis JH Abramson DH Ji X: Risk of Extraocular Extension in Eyes With Retinoblastoma Receiving Intravitreous Chemotherapy. *JAMA Ophthalmol.* 2017;135(12):1426–9. 10.1001/jamaophthalmol.2017.4600 29098285 PMC6583521

[32] Munier FL Soliman S Moulin AP: Profiling safety of intravitreal injections for retinoblastoma using an anti-reflux procedure and sterilisation of the needle track. *Br J Ophthalmol.* 2012;96(8):1084–7. 10.1136/bjophthalmol-2011-301016 22368262

[33] Kingston JE Hungerford JL Madreperla SA: Results of combined chemotherapy and radiotherapy for advanced intraocular retinoblastoma. *Arch Ophthalmol.* 1996;114(11):1339–43. 10.1001/archopht.1996.01100140539004 8906024

[34] Mendoza PR Grossniklaus HE: Therapeutic Options for Retinoblastoma. *Cancer Control.* 2016;23(2):99–109. 10.1177/107327481602300203 27218786

[35] Shields CL Mashayekhi A Au AK: The International Classification of Retinoblastoma predicts chemoreduction success. *Ophthalmology.* 2006;113(12):2276–80. 10.1016/j.ophtha.2006.06.018 16996605

[36] Shields CL Shields JA Meadows AT: Chemoreduction for retinoblastoma may prevent trilateral retinoblastoma. *J Clin Oncol.* 2000;18(1):236–7. 10.1200/JCO.2000.18.1.236 10623718

[37] Dunkel IJ Jubran RF Gururangan S: Trilateral retinoblastoma: potentially curable with intensive chemotherapy. *Pediatr Blood Cancer.* 2010;54(3):384–7. 10.1002/pbc.22336 19908299

[38] Ortiz MV Dunkel IJ: Retinoblastoma. *J Child Neurol.* 2016;31(2):227–36. 10.1177/0883073815587943 26023180

[39] Shields CL Mashayekhi A Sun H: Iodine 125 plaque radiotherapy as salvage treatment for retinoblastoma recurrence after chemoreduction in 84 tumors. *Ophthalmology.* 2006;113(11):2087–92. 10.1016/j.ophtha.2006.04.032 16949158

[40] Francis JH Barker CA Wolden SL: Salvage/adjuvant brachytherapy after ophthalmic artery chemosurgery for intraocular retinoblastoma. *Int J Radiat Oncol Biol Phys.* 2013;87(3):517–23. 10.1016/j.ijrobp.2013.06.2045 23953635 PMC4843130

[41] McLaughlin CC Wu XC Jemal A: Incidence of noncutaneous melanomas in the U.S. *Cancer.* 2005;103(5):1000–7. 10.1002/cncr.20866 15651058

[42] Collaborative Ocular Melanoma Study Group: Assessment of metastatic disease status at death in 435 patients with large choroidal melanoma in the Collaborative Ocular Melanoma Study (COMS): COMS report no. 15. *Arch Ophthalmol*.2001;119(5):670–6. 10.1001/archopht.119.5.670 11346394

[43] Singh AD Topham A: Survival rates with uveal melanoma in the United States: 1973–1997. *Ophthalmology.* 2003;110(5):962–5. 10.1016/S0161-6420(03)00077-0 12750098

[44] Diener-West M Reynolds SM Agugliaro DJ: Development of metastatic disease after enrollment in the COMS trials for treatment of choroidal melanoma: Collaborative Ocular Melanoma Study Group Report No. 26. *Arch Ophthalmol.* 2005;123(12):1639–43. 10.1001/archopht.123.12.1639 16344433

[45] Kujala E Mäkitie T Kivelä T: Very long-term prognosis of patients with malignant uveal melanoma. *Invest Ophthalmol Vis Sci.* 2003;44(11):4651–9. 10.1167/iovs.03-0538 14578381

[46] Diener-West M Earle JD Fine SL: The COMS randomized trial of iodine 125 brachytherapy for choroidal melanoma, III: initial mortality findings. COMS Report No. 18. *Arch Ophthalmol.* 2001;119(7):969–82. 10.1001/archopht.119.7.969 11448319

[47] Kim IK Lane AM Gragoudas ES: Survival in patients with presymptomatic diagnosis of metastatic uveal melanoma. *Arch Ophthalmol.* 2010;128(7):871–5. 10.1001/archophthalmol.2010.121 20625048

[48] Njauw CN Kim I Piris A: Germline *BAP1* inactivation is preferentially associated with metastatic ocular melanoma and cutaneous-ocular melanoma families. *PLoS One.* 2012;7(4):e35295. 10.1371/journal.pone.0035295 22545102 PMC3335872

[49] Collaborative Ocular Melanoma Study Group: Histopathologic characteristics of uveal melanomas in eyes enucleated from the Collaborative Ocular Melanoma Study. COMS report no. 6. *Am J Ophthalmol*.1998;125(6):745–66. 10.1016/S0002-9394(98)00040-3 9645714

[50] Augsburger JJ Corrêa ZM Trichopoulos N: Prognostic implications of cytopathologic classification of melanocytic uveal tumors evaluated by fine-needle aspiration biopsy. *Arq Bras Oftalmol.* 2013;76(2):72–9. 10.1590/S0004-27492013000200004 23828465

[51] Prescher G Bornfeld N Becher R: Nonrandom chromosomal abnormalities in primary uveal melanoma. *J Natl Cancer Inst.* 1990;82(22):1765–9. 10.1093/jnci/82.22.1765 2231772

[52] Helgadottir H Höiom V: The genetics of uveal melanoma: current insights. *Appl Clin Genet.* 2016;9:147–55. 10.2147/TACG.S69210 27660484 PMC5019476

[53] Daniels AB Lee JE MacConaill LE: High throughput mass spectrometry-based mutation profiling of primary uveal melanoma. *Invest Ophthalmol Vis Sci.* 2012;53(11):6991–6. 10.1167/iovs.12-10427 22977135 PMC3471553

[54] Harbour JW Onken MD Roberson ED: Frequent mutation of *BAP1* in metastasizing uveal melanomas. *Science.* 2010;330(6009):1410–3. 10.1126/science.1194472 21051595 PMC3087380

[55] Chang SH Worley LA Onken MD: Prognostic biomarkers in uveal melanoma: evidence for a stem cell-like phenotype associated with metastasis. *Melanoma Res.* 2008;18(3):191–200. 10.1097/CMR.0b013e3283005270 18477893

[56] Martin M Maßhöfer L Temming P: Exome sequencing identifies recurrent somatic mutations in *EIF1AX* and *SF3B1* in uveal melanoma with disomy 3. *Nat Genet.* 2013;45(8):933–6. 10.1038/ng.2674 23793026 PMC4307600

[57] Yavuzyigitoglu S Koopmans AE Verdijk RM: Uveal Melanomas with *SF3B1* Mutations: A Distinct Subclass Associated with Late-Onset Metastases. *Ophthalmology.* 2016;123(5):1118–28. 10.1016/j.ophtha.2016.01.023 26923342

[58] Field MG Harbour JW: Recent developments in prognostic and predictive testing in uveal melanoma. *Curr Opin Ophthalmol.* 2014;25(3):234–9. 10.1097/ICU.0000000000000051 24713608 PMC4467564

[59] Onken MD Worley LA Ehlers JP: Gene expression profiling in uveal melanoma reveals two molecular classes and predicts metastatic death. *Cancer Res.* 2004;64(20):7205–9. 10.1158/0008-5472.CAN-04-1750 15492234 PMC5407684

[60] Damato B Dopierala J Klaasen A: Multiplex ligation-dependent probe amplification of uveal melanoma: correlation with metastatic death. *Invest Ophthalmol Vis Sci.* 2009;50(7):3048–55. 10.1167/iovs.08-3165 19182252

[61] Harbour JW Chen R: The DecisionDx-UM Gene Expression Profile Test Provides Risk Stratification and Individualized Patient Care in Uveal Melanoma. *PLoS Curr.* 2013;5: pii: ecurrents.eogt.af8ba80fc776c8f1ce8f5dc485d4a618. 10.1371/currents.eogt.af8ba80fc776c8f1ce8f5dc485d4a618 23591547 PMC3625622

[62] Decatur CL Ong E Garg N: Driver Mutations in Uveal Melanoma: Associations With Gene Expression Profile and Patient Outcomes. *JAMA Ophthalmol.* 2016;134(7):728–33. 10.1001/jamaophthalmol.2016.0903 27123562 PMC4966162

[63] Shields CL Say EAT Hasanreisoglu M: Personalized Prognosis of Uveal Melanoma Based on Cytogenetic Profile in 1059 Patients over an 8-Year Period: The 2017 Harry S. Gradle Lecture. *Ophthalmology.* 2017;124(10):1523–31. 10.1016/j.ophtha.2017.04.003 28495150

[64] Field MG Decatur CL Kurtenbach S: PRAME as an Independent Biomarker for Metastasis in Uveal Melanoma. *Clin Cancer Res.* 2016;22(5):1234–42. 10.1158/1078-0432.CCR-15-2071 26933176 PMC4780366

[65] Field MG Durante MA Decatur CL: Epigenetic reprogramming and aberrant expression of PRAME are associated with increased metastatic risk in Class 1 and Class 2 uveal melanomas. *Oncotarget.* 2016;7(37):59209–19. 10.18632/oncotarget.10962 27486988 PMC5312306

[66] Reichstein D: New concepts in the molecular understanding of uveal melanoma. *Curr Opin Ophthalmol.* 2017;28(3):219–27. 10.1097/ICU.0000000000000366 28257297

[67] Gezgin G Luk SJ Cao J: PRAME as a Potential Target for Immunotherapy in Metastatic Uveal Melanoma. *JAMA Ophthalmol.* 2017;135(6):541–9. 10.1001/jamaophthalmol.2017.0729 28448663 PMC5509351

[68] Kim RS Chevez-Barrios P Bretana ME: Histopathologic Analysis of Transvitreal Fine Needle Aspiration Biopsy Needle Tracts for Uveal Melanoma. *Am J Ophthalmol.* 2017;174:9–16. 10.1016/j.ajo.2016.10.019 27818205

[69] Shields CL Ganguly A Materin MA: Chromosome 3 analysis of uveal melanoma using fine-needle aspiration biopsy at the time of plaque radiotherapy in 140 consecutive cases. *Trans Am Ophthalmol Soc.* 2007;105:43–52; discussion 52–3. 18427593 PMC2258107

[70] Chang MY McCannel TA: Comparison of uveal melanoma cytopathologic sample retrieval in trans-scleral versus vitrectomy-assisted transvitreal fine needle aspiration biopsy. *Br J Ophthalmol.* 2014;98(12):1654–8. 10.1136/bjophthalmol-2014-305181 24997179

[71] Sellam A Desjardins L Barnhill R: Fine Needle Aspiration Biopsy in Uveal Melanoma: Technique, Complications, and Outcomes. *Am J Ophthalmol.* 2016;162:28–34.e1. 10.1016/j.ajo.2015.11.005 26556006

[72] Singh AD Medina CA Singh N: Fine-needle aspiration biopsy of uveal melanoma: outcomes and complications. *Br J Ophthalmol.* 2016;100(4):456–62. 10.1136/bjophthalmol-2015-306921 26231747

[73] Correa ZM Augsburger JJ: Sufficiency of FNAB aspirates of posterior uveal melanoma for cytologic versus GEP classification in 159 patients, and relative prognostic significance of these classifications. *Graefes Arch Clin Exp Ophthalmol.* 2014;252(1):131–5. 10.1007/s00417-013-2515-0 24270974 PMC3889697

[74] Jampol LM Moy CS Murray TG: The COMS randomized trial of iodine 125 brachytherapy for choroidal melanoma: IV. Local treatment failure and enucleation in the first 5 years after brachytherapy. COMS report no. 19. *Ophthalmology.* 2002;109(12):2197–206. 10.1016/S0161-6420(02)01277-0 12466159

[75] Nag S Quivey JM Earle JD: The American Brachytherapy Society recommendations for brachytherapy of uveal melanomas. *Int J Radiat Oncol Biol Phys.* 2003;56(2):544–55. 10.1016/S0360-3016(03)00006-3 12738332

[76] Collaborative Ocular Melanoma Study Group: The COMS randomized trial of iodine 125 brachytherapy for choroidal melanoma: V. Twelve-year mortality rates and prognostic factors: COMS report No. 28. *Arch Ophthalmol.* 2006;124(12):1684–93. 10.1001/archopht.124.12.1684 17159027

[77] Ophthalmic Oncology Task Force: Local Recurrence Significantly Increases the Risk of Metastatic Uveal Melanoma. *Ophthalmology.* 2016;123(1):86–91. 10.1016/j.ophtha.2015.09.014 26505803

[78] Harbour JW Char DH Kroll S: Metastatic risk for distinct patterns of postirradiation local recurrence of posterior uveal melanoma. *Ophthalmology.* 1997;104(11):1785–92; discussion 1792–3. 10.1016/S0161-6420(97)30025-6 9373108

[79] Berry JL Dandapani SV Stevanovic M: Outcomes of choroidal melanomas treated with eye physics: a 20-year review. *JAMA Ophthalmol.* 2013;131(11):1435–42. 10.1001/jamaophthalmol.2013.4422 24008431

[80] Tann AW Teh BS Scarboro SB: Early outcomes of uveal melanoma treated with intraoperative ultrasound guided brachytherapy using custom built plaques. *Pract Radiat Oncol.* 2017;7(4):e275–e282. 10.1016/j.prro.2017.01.002 28377140

[81] Astrahan MA Luxton G Pu Q: Conformal episcleral plaque therapy. *Int J Radiat Oncol Biol Phys.* 1997;39(2):505–19. 10.1016/S0360-3016(97)00118-1 9308957

[82] Astrahan MA Luxton G Jozsef G: Optimization of ^125^I ophthalmic plaque brachytherapy. *Med Phys.* 1990;17(6):1053–7. 10.1118/1.596585 2280735

[83] Harbour JW Murray TG Byrne SF: Intraoperative echographic localization of iodine 125 episcleral radioactive plaques for posterior uveal melanoma. *Retina.* 1996;16(2):129–34. 8724957 10.1097/00006982-199616020-00008

[84] Tabandeh H Chaudhry NA Murray TG: Intraoperative echographic localization of iodine-125 episcleral plaque for brachytherapy of choroidal melanoma. *Am J Ophthalmol.* 2000;129(2):199–204. 10.1016/S0002-9394(99)00315-3 10682973

[85] Finger PT: Intraoperative echographic localization of iodine-125 episcleral plaque for brachytherapy of choroidal melanoma. *Am J Ophthalmol.* 2000;130(4):539–40. 10.1016/S0002-9394(00)00549-3 11183561

[86] Aziz HA Al Zahrani YA Bena J: Episcleral brachytherapy of uveal melanoma: role of intraoperative echographic confirmation. *Br J Ophthalmol.* 2017;101(6):747–51. 10.1136/bjophthalmol-2016-309153 27574179

[87] Astrahan MA Luxton G Jozsef G: An interactive treatment planning system for ophthalmic plaque radiotherapy. *Int J Radiat Oncol Biol Phys.* 1990;18(3):679–87. 10.1016/0360-3016(90)90077-W 2318702

[88] Wang Z Nabhan M Schild SE: Charged particle radiation therapy for uveal melanoma: a systematic review and meta-analysis. *Int J Radiat Oncol Biol Phys.* 2013;86(1):18–26. 10.1016/j.ijrobp.2012.08.026 23040219

[89] Mishra KK Daftari IK: Proton therapy for the management of uveal melanoma and other ocular tumors. *Chin Clin Oncol.* 2016;5(4):50. 10.21037/cco.2016.07.06 27558251

[90] Melia BM Abramson DH Albert DM: Collaborative ocular melanoma study (COMS) randomized trial of I-125 brachytherapy for medium choroidal melanoma. I. Visual acuity after 3 years COMS report no. 16. *Ophthalmology.* 2001;108(2):348–66. 10.1016/S0161-6420(00)00526-1 11158813

[91] Corrêa ZM Augsburger JJ: Independent Prognostic Significance of Gene Expression Profile Class and Largest Basal Diameter of Posterior Uveal Melanomas. *Am J Ophthalmol.* 2016;162:20–27.e1. 10.1016/j.ajo.2015.11.019 26596399

[92] Walter SD Chao DL Feuer W: Prognostic Implications of Tumor Diameter in Association With Gene Expression Profile for Uveal Melanoma. *JAMA Ophthalmol.* 2016;134(7):734–40. 10.1001/jamaophthalmol.2016.0913 27123792 PMC4966166

[93] Schefler A Berry D Seider M: Ocular Oncology Study Consortium Report 3: Baseline clinical features and relationship to GEP Class. *Association for Research in Vision and Ophthalmology.*Baltimore, MD.2017.

[94] Chew AL Spilsbury K Isaacs TW: Survival from uveal melanoma in Western Australia 1981–2005. *Clin Exp Ophthalmol.* 2015;43(5):422–8. 10.1111/ceo.12490 25556534

[95] Kaiserman I Anteby I Chowers I: Post-brachytherapy initial tumour regression rate correlates with metastatic spread in posterior uveal melanoma. *Br J Ophthalmol.* 2004;88(7):892–5. 10.1136/bjo.2003.036285 15205232 PMC1772205

[96] Shields CL Bianciotto C Rudich D: Regression of uveal melanoma after plaque radiotherapy and thermotherapy based on chromosome 3 status. *Retina.* 2008;28(9):1289–95. 10.1097/IAE.0b013e31817f7b3e 18628721

[97] Gupta K McCannel CA Kamrava M: Tumor-height regression rate after brachytherapy between choroidal melanoma gene expression profile classes: effect of controlling for tumor height. *Graefes Arch Clin Exp Ophthalmol.* 2016;254(7):1371–8. 10.1007/s00417-016-3305-2 26907932

[98] Corrêa ZM Augsburger JJ: Relationship between rate of posterior uveal melanoma flattening following plaque radiotherapy and gene expression profile class of tumor cells. *Invest Ophthalmol Vis Sci.* 2014;55(1):556–9. 10.1167/iovs.13-13381 24408979

[99] Rao RC Khan M Badiyan SN: Gene expression profiling and regression rate of irradiated uveal melanomas. *Ophthalmic Surg Lasers Imaging Retina.* 2015;46(3):333–7. 10.3928/23258160-20150323-06 25856819 PMC4398963

[100] Mruthyunjaya P Seider MI Stinnett S: Association between Tumor Regression Rate and Gene Expression Profile after Iodine 125 Plaque Radiotherapy for Uveal Melanoma. *Ophthalmology.* 2017;124(10):1532–9. 10.1016/j.ophtha.2017.04.013 28549517

[101] Landreville S Agapova OA Matatall KA: Histone deacetylase inhibitors induce growth arrest and differentiation in uveal melanoma. *Clin Cancer Res.* 2012;18(2):408–16. 10.1158/1078-0432.CCR-11-0946 22038994 PMC3261307

[102] Chen X Wu Q Tan L: Combined PKC and MEK inhibition in uveal melanoma with GNAQ and GNA11 mutations. *Oncogene.* 2014;33(39):4724–34. 10.1038/onc.2013.418 24141786 PMC4524511

[103] Nowis D Makowski M Stoklosa T: Direct tumor damage mechanisms of photodynamic therapy. *Acta Biochim Pol.* 2005;52(2):339–52. 15990919

